# Health Tract, No. 319

**Published:** 1868-03

**Authors:** 


### HEALTH TRACT, No. 319.

### POVERTY AND LONGEVITY.

NEITHER poverty, hardships nor exposures tend to lengthen life; they always shorten the average of life by many years, except in those few cases where there are counteracting conditions. In the great city of New York where perhaps full half of the population are steeped in poverty and destitution of one hundred children dying, only ten are the children of the rich—of the well-to-do. In the filthiest parts of the city ten times as many die as along Fifth Avenue and the two streets on either side of an equal number of the population. M. Benoiston, Casper, Dr. Major, and others, after very minute investigations state, "The value of life is less among the empoverished, than among the rich; thus of an equal number of infants of the same age. double the number die of the poorer than of the wealthier class; where there is the greatest misery, there is the greatest mortality." Casper states that in Berlin the mean duration of life among the better classes is fifty years, but among the paupers thirty two years only. Of one thousand infants of the nobility of Germany, fifty-seven die in the first five years; of the same number taken from indigent families, there died in the same time three hundred and forty-five and that while half the poor reach the thirty-second year only, half the noble live twenty years longer. The Queen of England has had nearly a dozen children and every one of them has grown up to man's estate in vigorous health. The pensioned Noble and the paupered inhabitant of the work-house live indefinitely long the world over, for they are alike relieved of the corroding care for to-morrow's bread; they both live tidily, the latter from compulsion, the former from choice.

The lessons from these facts are of great personal practical value to every household in the land.

First. The well-to-do can command the comforts, conveniences and tidiness of life; they can command the best ways of doing things and thus economize time, labor and care; they have regular habits of eating; they have their full share of regular sleep; they have the best of food, well cooked; they have abundance of rest, and are exempt from exposures to the extremes of heat and cold; are warmly clad in winter, and in summer live in well aired and spacious apartments,—their intelligence leading them to arrange that these things shall be brought about.

Second. A practical knowledge of the laws of our being leads to large results in the direction of human happiness, health and enjoyment.

QUERY.

Do not those parents commit a crime against society who fail to instruct their daughters in the duties of the household and of maternity? To know how to keep a tidy house, and well aired apartments; to know how to select the best kinds of food; to know how to prepare them in the best manner; how to watch over helpless infancy—these are first things.

